# Early Surveillance Endoscopy Should Be Performed Selectively After Transanal Endoscopic Microsurgery for Rectal Lesions

**DOI:** 10.7759/cureus.60554

**Published:** 2024-05-18

**Authors:** James R Holden, Garrett Johnson, David Hochman, Eric Hyun, Ramzi M Helewa

**Affiliations:** 1 Surgery, University of Saskatchewan, Saskatoon, CAN; 2 Surgery, University of Manitoba, Winnipeg, CAN

**Keywords:** rectal cancer surveillance, endoscopy, minimally invasive surgery, rectal cancer, rectal polyps, transanal endoscopic microsurgery, transanal endoscopic surgery

## Abstract

Introduction

Local recurrence (LR) rates after transanal endoscopic microsurgery (TEM) are unclear, and the utility of early postoperative surveillance for low-risk lesions is unknown. This study aimed to define LR after TEM for benign polyps and invasive adenocarcinoma, describe risk factors for LR, and evaluate the utility of early surveillance endoscopy.

Methods

This retrospective cohort study was conducted at two hospitals in Winnipeg, Manitoba, Canada. Adult patients who underwent TEM between 2009 and 2020 were evaluated for inclusion. The primary outcome was the rate of LR on surveillance endoscopy. Other outcomes included risk factors for LR and diagnostic yield of surveillance endoscopy.

Results

Among 357 patients who underwent TEM for benign polyps, LR was 10.5% (95% confidence interval (CI) 5.8-15.2) at three years. Positive margin was correlated with LR on multivariate analysis (hazard ratio (HR) 8.01, 95% CI 2.78-23.08). TEM defect closure was associated with lower LR on multivariate analysis (HR 0.19, 95% CI 0.06-0.59). Among 124 patients who underwent TEM for rectal adenocarcinoma, LR was 15.0% (95% CI 6.0-24.0) at three years. The first surveillance endoscopy had a 1.4% yield for low-risk patients (benign lesion, negative margins, and closed TEM defect) and 6.9% for all others.

Conclusions

LR at three years after TEM was 10.5% for benign polyps and 15.0% for adenocarcinomas. Early surveillance endoscopy can be considered low yield in some patients after TEM, which can be informative for shared decision-making regarding whether to proceed with early endoscopy in a low-risk subgroup of patients.

## Introduction

Endoscopic examination after transanal endoscopic microsurgery (TEM) is important to assess for local recurrence (LR), but the ideal timing of surveillance interval is unclear. For early rectal adenocarcinomas, the American Society of Colon and Rectal Surgeons (ASCRS) guidelines provide a weak recommendation for proctoscopy every six months for three to five years after excision [1]. Guidelines from the National Comprehensive Cancer Network (NCCN) and United States Multi-Society Services Task Force (USMSTF) suggest surveillance regimens that differ in frequency, duration, and the use of adjunctive technologies, such as endorectal ultrasound and contrast magnetic resonance imaging (MRI) [1,2]. Furthermore, there is a paucity of evidence to guide recommendations for endoscopic surveillance after TEM for non-invasive polyps. Many surgeons extrapolate from endoscopy guidelines that recommend short-interval endoscopic evaluations (<1 year) for polyps removed in a piecemeal fashion [3]. However, TEM will often result in lesions that are removed completely without fragmentation. For those lesions with negative margins, the optimal surveillance interval is unknown. Our current local practice is to perform an initial flexible sigmoidoscopy approximately six months after TEM and a full colonoscopy three years postoperatively. Identifying the preferred surveillance regimen for such patients is key because repeat endoscopies are associated with a significant cost and resource burden to the healthcare system [4-6].

Since the introduction of TEM, estimates of LR have varied considerably, depending on the lesion type (including both benign and malignant lesions), technical expertise, chemotherapy and/or radiation treatment, and the surveillance regimen used [7]. Authors have reported LR as low as 2.4% at three years, or as high as 35% at five years [8,9]. As TEM has become more widespread and expertise has increased, the applicability of older LR estimates is unclear. Furthermore, the clinical benefit of short-interval surveillance after TEM is unknown. Therefore, the objectives of this study are to identify LR rates after TEM with a focus on pathologic and surgical risk factors. An additional objective is to determine the utility and yield of early surveillance endoscopy, particularly with respect to low-risk patient groups.

## Materials and methods

Study design and research ethics board approval

This is a retrospective cohort study of patients from two high-volume TEM hospitals in Winnipeg, Manitoba, Canada (St. Boniface Hospital and Victoria General Hospital). This study was approved by the University of Manitoba’s Health Research Ethics Board (approval no. HS25083 (H2021:272)).

Patient cohort

All adult patients (≥18 years old) who underwent TEM in Winnipeg between 2009 and 2020 were evaluated. All TEM was performed by two colorectal surgeons during this time period, one beginning in 2009 and the other in 2016. Patients were categorized as either benign neoplastic polyps or invasive adenocarcinomas. In the benign group, patients with a non-neoplastic process (i.e., fistula-in-ano, rectovaginal fistula, and prolapsing rectal mucosa) were excluded. In the malignant group, patients with a diagnosis of neuroendocrine tumor, gastrointestinal stromal tumor, squamous cell carcinoma, lymphoma, or other non-adenocarcinoma malignant lesions were excluded. Patients who underwent immediate radical resection without any surveillance period post-TEM were also excluded. In both groups, patients who did not have a documented follow-up endoscopy of the TEM site were excluded.

Outcomes

Baseline demographic and clinical characteristics were collected from a combination of surgeon office charts and electronic medical record data. The following variables were collected for all patients: age, sex, TEM defect closure, lesion location, subsequent radical surgery, date of surgery, date of surveillance endoscopies, LR, and distant recurrence. For the benign patient group, the following pathology data were collected: lesion type, lesion histology, margin status, lesion diameter, and presence of high-grade dysplasia (HGD). Lesion diameter was measured from pathology report data. For the malignant patient group, the following pathology data were collected: lesion type, pathologic T stage, depth of invasion (if pT1/ypT1 lesion), margin status, differentiation, lymphovascular invasion (LVI), perineural invasion (PNI), mucinous features, tumor budding, and lesion diameter. Lesion location (i.e., anterior, posterior, left, and right) was determined from patient operative reports. Lesion diameter was measured from pathology reports, and the size of the invasive cancer component of the lesion was also measured when reported. All patients underwent TEM specimen analysis by a trained pathologist. LR was defined as the presence of any biopsy-verified benign or malignant neoplastic tissue at the previous TEM site found on endoscopic examination. Yield was determined using the proportion of surveillance endoscopy procedures where LR was detected. The follow-up period was defined as the time between the index TEM procedure and the last available endoscopy report or occurrence of any LR event.

Statistical analysis

Time to LR was analyzed via univariable Kaplan-Meier plots, with pairwise comparisons made using log-rank (Mantel-Cox) tests. Patient observations were censored on the date of the last examination. Univariable and multivariable Cox proportional hazards regression models were used to identify risk factors associated with earlier recurrence. Benign polyps and invasive adenocarcinoma lesions were analyzed separately. All sets of variables applicable to the lesion type were analyzed. The predictor variables used for all lesions were age, sex, lesion diameter, defect closure, and margin status. For adenocarcinomas, differentiation, pathologic T stage, LVI, tumor budding, and mucinous features were also predictor variables. For neoplastic polyps, the degree of dysplasia was also used. Patients with missing data had those items excluded from analysis and are indicated alongside results where appropriate. Data were expressed in terms of hazard ratios, with 95% confidence intervals and relative p-values reported. Explanatory variables with univariable p≤0.20 were included in a multivariable analysis. Sensitivity analyses were performed for multi-categorical variables when some components met the p ≤ 0.20 inclusion cutoff and others did not (e.g., benign polyp histology) to determine whether their inclusion affected overall multivariable model fit. Statistical significance was determined at p = 0.05. Statistical analysis was performed using IBM SPSS Statistics for Windows, version 27.0 (released 2020, IBM Corp., Armonk, NY).

## Results

A total of 373 TEM procedures for benign indications were assessed for inclusion during the study period. Three hundred fifty-seven (357) met the inclusion criteria for the benign polyp group, while 16 were excluded (11 with non-polyp pathology, four with incomplete operative or pathology data, and one aborted procedure). A total of 166 TEM procedures for malignancy were evaluated, and 124 were included in the final analysis. Twenty-eight (28) patients were excluded because of non-adenocarcinoma pathology (neuroendocrine tumor, gastrointestinal stromal tumor, or squamous cell carcinoma), while 14 were excluded because of incomplete operative or pathology data. PNI was excluded from statistical analyses, as this was not reported for most specimens. Depth of invasion was collected but not analyzed as it was reported infrequently and with non-comparable terminology (e.g., inconsistent use of sm/Kikuchi classification, Haggitt classification, or depth of invasion in micrometers) [10,11].

Among patients with benign polyps, the mean age was 67 years, and 44.3% of the patients were female (Table 1). The TEM defect was sutured closed in 241 (67.5%) patients. The final pathology demonstrated adenomatous polyps in 349 (97.8%) patients, with 144 (40.3%) lesions showing HGD. Negative microscopic margins were achieved in 314 (88.0%) cases. In the adenocarcinoma group, the mean patient age was 68 years and 51 (41.1%) patients were female. TEM defect closure occurred in 86 (69.4%) of these cases. The majority of lesions (n = 100, 80.6%) were pT1/ypT1 on the final pathology.

**Table 1:** Patient demographics and lesion characteristics Value indicates n (%) except where otherwise indicated. †No location data available for 1 cancer and 11 adenomas. Not reported in *27 specimens, **112 specimens, ***one specimen, ****63 specimens. LVI, lymphovascular invasion; NOS, not otherwise specified; PNI, perineural invasion; SSA, sessile serrated adenoma; TSA, traditional serrated adenoma; HGD, high-grade dysplasia.

Demographics	Benign polyps (n = 357)	Adenocarcinomas (n = 124)
Mean age, years	67 (±SD 11.4)	68 (±SD 11.9)
Total LRs	22 (6.2%)	12 (9.7%)
Median follow-up, months	11.0 (IQR 6.5-23.6)	14.2 (IQR 6.8-37.9)
Sex, females	158 (44.3%)	51 (41.1%)
Mean lesion diameter, cm	3.55 (±SD 1.07)	2.75 (±SD 2.06)
Defect closure	241 (67.5%)	86 (69.4%)
Margin		
Negative	314 (88.0%)	107 (86.3%)
Positive	34 (9.5%)	13 (10.5%)
Unclear	9 (2.5%)	4 (3.2%)
Polyp histology		
Unspecified	3 (0.8%)	-
Hyperplastic	5 (1.4%)	-
TSA	21 (5.9%)	-
SSA	6 (1.7%)	-
Tubular adenoma	38 (10.6%)	-
Tubulovillous adenoma	202 (56.6%)	-
Villous adenoma	78 (21.8%)	-
Villous with SSA features	4 (1.1%)	-
HGD	144 (40.3%)	-
pT stage		
1	-	100 (80.6%)
2	-	15 (12.1%)
3	-	9 (7.3%)
Differentiation		
Well	-	61 (49.2%)
Moderate	-	33 (26.6%)
Poor	-	9 (7.3%)
LVI*	-	10 (8.1%)
PNI**	-	1 (0.8%)
Tumor budding***	-	12 (9.7%)
Mucinous****	-	7 (5.6%)

**Figure 1:**
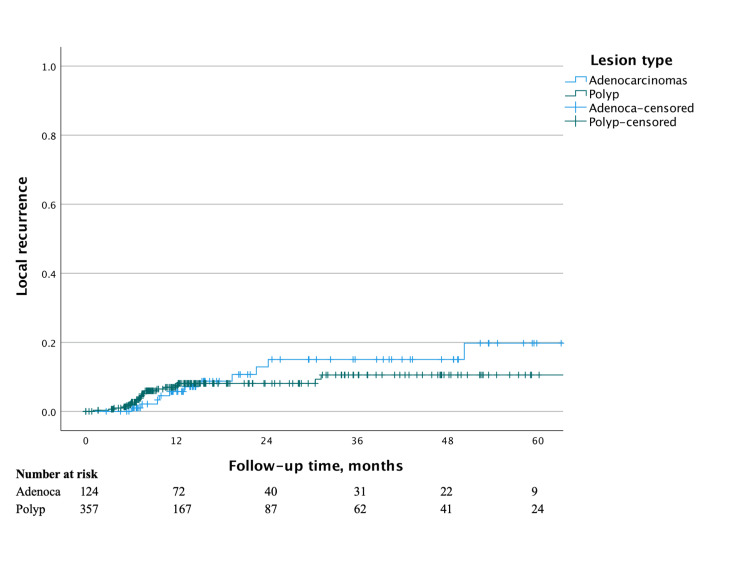
Kaplan-Meier survival curve for local recurrence after transanal endoscopic microsurgery for benign polyps and adenocarcinomas

For benign polyps, the Kaplan-Meier estimate of LR was 7.5% (95% CI 4.2-10.8) at one year, 8.1% (95% CI 4.6-11.6) at two years, and 10.5% (95% CI 5.8-15.2) at three years postoperatively (Figure 1). The median follow-up period was 11 months. Forty (40) patients did not undergo surveillance endoscopy. When recurrence did occur, one patient developed adenocarcinoma while all others were non-invasive adenomatous tissue. A positive margin on the final pathology was associated with LR for benign polyps on both univariate (hazard ratio (HR) 8.02, 95% confidence interval (CI) 3.27-19.67, p < 0.001) and multivariate (HR 8.01, 95% CI 2.78-23.08, p < 0.001) analysis (Table 2). TEM defect closure demonstrated a statistically significant negative correlation with LR on both univariate (HR 0.18, 95% CI 0.07-0.47, p < 0.001) and multivariate (HR 0.19, 95% CI 0.06-0.59, p = 0.004) analysis.

**Table 2:** Predictors of local recurrence after transanal endoscopic microsurgery for benign polyps NOS, not otherwise specified; SSA, sessile serrated adenoma; TSA, traditional serrated adenoma; HGD, high-grade dysplasia. p < 0.05 considered statistically significant.

Variable	Univariable analysis		Multivariable analysis	
	Hazard ratio	p-value	Hazard ratio	p-value
Age	1.02 (0.98-1.06)	0.46		
Female sex	1.37 (0.89-2.09)	0.15	1.96 (0.70-5.47)	0.20
Polyp histology				
Hyperplastic	ref		ref	
SSA	0.60 (0.04-9.73)	0.72	1.45 (0.07-28.49)	0.81
TSA	0.64 (0.06-7.08)	0.72	0.08 (0.00-1.59)	0.10
Tubular adenoma	0.27 (0.02-2.95)	0.28	0.28 (0.02-3.29)	0.31
Tubulovillous adenoma	0.19 (0.02-1.53)	0.12	0.12 (0.01-1.25)	0.07
Villous adenoma	0.58 (0.07-4.72)	0.61	0.50 (0.05-5.31)	0.57
Adenoma NOS	1.70 (0.11-27.49)	0.71	1.86 (0.11-30.62)	0.67
Positive margin	8.02 (3.27-19.67)	<0.001	8.01 (2.78-23.08)	<0.001
Lesion diameter	1.17 (0.99-1.39)	0.06	1.00 (0.83-1.21)	0.99
HGD	0.82 (0.34-1.94)	0.65		
Defect closure	0.18 (0.07-0.47)	<0.001	0.19 (0.062-0.588)	0.004

For patients with invasive adenocarcinoma, Kaplan-Meier analysis showed that LR was 5.7% (95% CI 0.8-10.6) at one year, 12.9% (95% CI 4.7-20.1) at two years, and 15.0% (95% CI 6.0-24.0) at three years post-procedure. The median follow-up period was 14.2 months, with 18 patients failing to undergo any surveillance endoscopy. T3 pathologic tumor stage was associated with a higher risk of LR on multivariate (HR 7.86, 95% CI 1.00-61.84, p = 0.05) analysis only (Table 3). The presence of mucinous features on pathology was strongly associated with LR on both univariate (HR 22.23, 95% CI 4.27-115.77, p < 0.001) and multivariate (HR 37.96, 95% CI 2.87-502.38, p = 0.006) analysis. Three instances of local recurrence were comprised of non-invasive adenomatous tissue, while the remaining recurrences were all adenocarcinoma. Eight patients in the malignant group (6.5%) developed distant metastatic disease, one of whom also had LR. Three patients received neoadjuvant chemotherapy with FOLFOX or CAPOX, and one patient received neoadjuvant radiation, with none of the patients showing evidence of recurrent disease on surveillance.

**Table 3:** Predictors of local recurrence after transanal endoscopic microsurgery for adenocarcinomas LVI, lymphovascular invasion. p < 0.05 considered statistically significant.

Variable	Univariable analysis		Multivariable analysis	
	Hazard ratio	p-value	Hazard ratio	p-value
Age	1.04 (0.99-1.10)	0.13	0.98 (0.92-1.05)	0.62
Female sex	1.30 (0.41-4.15)	0.66		
pT stage				
T1	ref		ref	
T2	1.99 (0.42-9.43)	0.39	0.40 (0.03-4.99)	0.47
T3	4.63 (0.96-22.38)	0.06	7.86 (1.00-61.84)	0.05
Differentiation				
Well	ref	-	-	-
Moderate	0.82 (0.19-3.51)	0.79	-	-
Poor	3.23 (0.37-28.32)	0.29	-	-
Positive margin	22.13 (0.00-4.2121E+6)	0.61	-	-
Lesion diameter	1.26 (0.99-1.60)	0.06	1.15 (0.75-1.77)	0.53
Defect closure	0.37 (0.12-1.15)	0.09	0.53 (0.08-3.64)	0.52
LVI	0.05 (0.00-8.07E+12)	0.86	-	-
Tumor budding	2.56 (0.25-25.96)	0.43	-	-
Mucinous	22.23 (4.27-115.77)	<0.001	37.96 (2.87-502.38)	0.006

**Figure 2:**
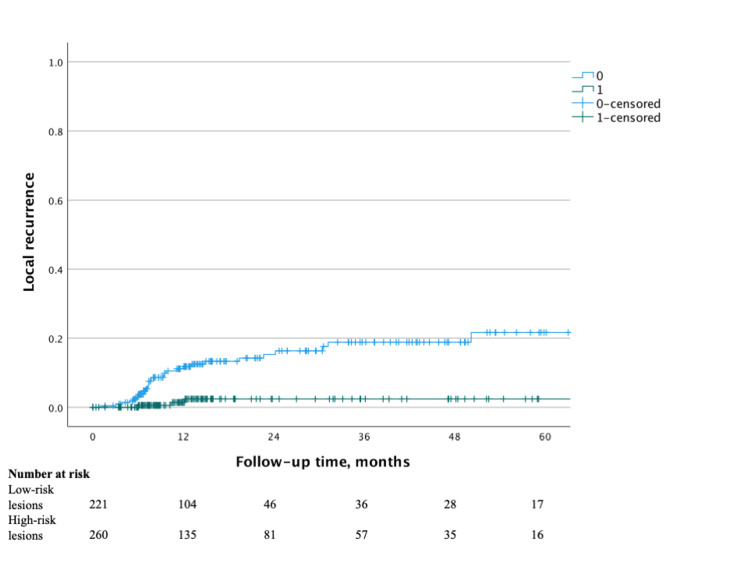
Kaplan-Meier survival curve for local recurrence after transanal endoscopic microsurgery for low-risk lesions (benign polyps resected with negative margins and closed surgical defects) and high-risk lesions

The measured median time to the first surveillance endoscopy after the index TEM procedure was 6.9 (IQR 5.9-7.7) months, and the yield of that scope in detecting LR was 4.4%. Given the lower rates of LR associated with benign polyps with negative margin status and closed TEM defect, a low-risk patient group with these characteristics was specifically evaluated against all other patients, who were designated as a high-risk patient group. The low-risk patient group included 221 patients. The median time to first surveillance endoscopy in the low-risk patient group was 7.0 (IQR 6.3-7.9) months, and the yield of that endoscopy was 1.4% (Figure 2). When these patients did have LR, all patients presented with benign disease. All patients were successfully managed with endoscopic or repeat TEM excision, and none had a repeat recurrence during the study period. In the high-risk group of 260 patients, the yield of initial surveillance endoscopy was 6.9% at a median time of 6.7 (IQR 5.3-7.7) months postoperatively.

## Discussion

Transanal local excision techniques have increased in popularity during recent decades, but surveillance guidelines include only weak recommendations for malignant lesions and are entirely absent for benign polyps. Given the risks to patients and the burdens to healthcare systems associated with potentially unnecessary repeat endoscopy, identifying risk factors for LR to help inform surveillance decision-making is critical. Here, we describe the three-year LR rates of 10.5% for benign polyps and 15.0% for invasive adenocarcinoma after TEM. Furthermore, we have identified positive margin as a risk factor and defect closure as a protective factor for LR among benign polyps, while T3 pathologic stage and mucinous features are risk factors among adenocarcinoma patients.

A post-TEM surveillance regimen based on patient risk factors has not yet been described, and current practices are potentially resulting in unnecessary risks to patients and costs to the healthcare system. We have identified a low-risk patient group who have benign rectal polyps that have been excised with negative margins and undergone TEM defect closure. In this group of patients, the likelihood of finding LR at an initial surveillance endoscopy six months postoperatively is very low. In the rare instances when recurrence was detected, it was always a benign disease, and it was managed without the need for radical surgery. This indicates that judicious selection of an endoscopic surveillance regimen tailored to a patient’s specific risk may safely reduce endoscopy needs without affecting patient prognosis. Furthermore, consideration could be made to follow routine national polyp surveillance guidelines for this patient group.

Strengths of this study include the large size of its patient cohort and robust statistical analysis of risk factors for LR, separately considering benign polyp and invasive adenocarcinoma groups. To our knowledge, this group of 357 patients is the largest published TEM cohort of benign lesions where LR was examined. This study is also the first to examine the yield of early surveillance endoscopy after TEM.

Several smaller studies have previously examined LR after TEM for benign adenomas. Allaix and colleagues published on patients with a rectal adenoma of at least 3 cm diameter, reporting a 5.6% LR rate and an association with positive margin status [12]. Another study of 75 patients with rectal adenoma showed an LR rate of 15% and demonstrated a strong association between LR and positive margin status [13]. Ganai et al. demonstrated a 15% LR rate after TEM for rectal adenomas, demonstrating an association with HGD but not margin status [9]. In relation to these data, our own LR rate is comparable, and the impact of positive margin status is further emphasized by our work here.

TEM outcomes in relation to the closure of the surgical defect have been examined in one randomized control trial of Canadian patients, although only short-term postoperative complications were evaluated in this work, rather than local recurrence [14]. In our results, sutured closure of the TEM defect was associated with a lower risk of LR. There is significant potential for confounding here, as many of these patients may have had larger lesions that made defect closure non-feasible. Our local practice is to routinely suture the TEM defect closed except in the cases of a large defect where the closure would be under significant tension or a very distal defect close to the dentate line. Interestingly, the lesion diameter was not correlated with LR in our study. Although specimen handling and processing prior to pathologic analysis may affect lesion diameter measurements, this represents a difference between our own findings and those of several other published works [9,12].

Analysis of the literature regarding LR after TEM for rectal adenocarcinoma confirms the influence of tumor depth of invasion into the rectal wall. Two separate studies have demonstrated a steady increase in LR rates between pT1, pT2, and pT3 disease, with tumor depth of invasion and T stage representing independent risk factors for LR [7,15]. Ganai et al. showed an overall LR rate of 15% for rectal cancers treated with TEM and also found that the depth of tumor invasion was a risk factor. Among potentially adverse histopathologic features, LVI has previously shown a demonstrable link to the LR rate [7,15]. However, mucinous pathologic features have seldom been reported in the literature when examining LR after TEM. Our work here is the first to clearly establish this link. Further research will be required to evaluate any effects of differential surveillance regimens on overall prognosis and to determine whether there are higher-risk groups of patients who would benefit from more intensive surveillance.

It must be acknowledged that there are several limitations to this work. There is potential bias related to the retrospective nature of the data and its origin from two surgeons working at a tertiary medical center, which may affect the generalizability of these results. In addition, long-term surveillance data for a significant number of patients are unknown. Where information was not reported, it is likely that patients underwent follow-up endoscopy with their original referring endoscopist. However, we are unable to capture this data. Despite the size of the patient population examined, this study group may have been underpowered to detect certain variables correlated with LR after TEM. Lastly, the heterogeneity of the adenocarcinoma patient population group is another potential drawback. The study includes some patients who underwent neoadjuvant therapy as part of a clinical trial, which may have led to stage migration. It also includes several patients with T2 or T3 rectal cancers who either were not candidates for radical surgery or refused it, introducing the potential for selection bias. While we are able to conclude that the six-month surveillance endoscopy is low yield in a low-risk subset of patients with fully resected adenomas, this study is unable to identify at what interval would be a more appropriate timeframe. To address all of these limitations, future multicenter prospective research is needed.

## Conclusions

We have identified three-year LR rates of 10.5% for benign polyps and 15.0% for adenocarcinoma. This study is the first to demonstrate mucinous tumor histology as a risk factor for LR. The yield of early surveillance endoscopy is low following TEM for benign polyps with negative margins and a closed defect. Our data suggest that in carefully selected patients, delay of this six-month surveillance endoscopy could be considered. These data could be particularly important in resource-constrained settings where endoscopy is a precious resource.
